# A Unification of Nanotopography and Extracellular Matrix in Electrospun Scaffolds for Bioengineered Hepatic Models

**DOI:** 10.1021/acsabm.3c00032

**Published:** 2023-06-07

**Authors:** Yunxi Gao, Thomas S. R. Bate, Anthony Callanan

**Affiliations:** Institute of Bioengineering, School of Engineering, The University of Edinburgh, Edinburgh EH10 SHF, United Kingdom

**Keywords:** electrospinning, decellularized extracellular matrix, topography, scaffolds, liver tissue engineering

## Abstract

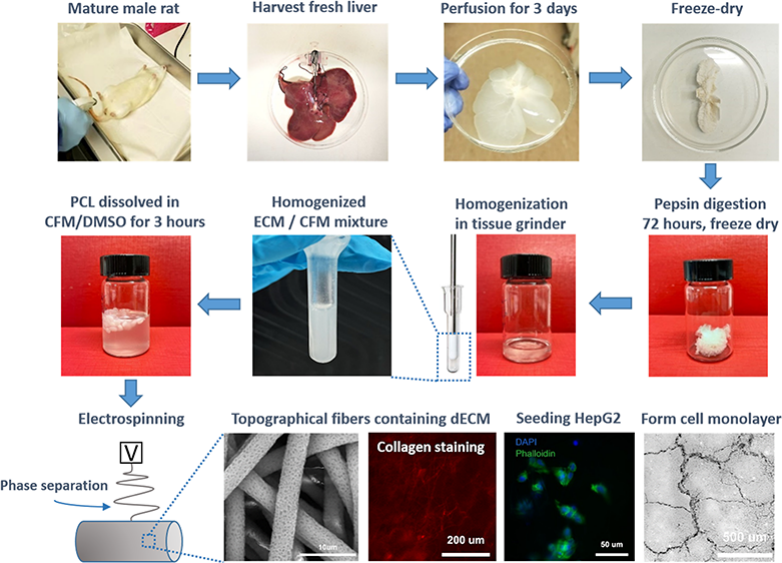

Donor liver shortage is a crucial global public health problem as whole-organ transplantation is the only definitive cure for liver disease. Liver tissue engineering aims to reproduce or restore function through in vitro tissue constructs, which may lead to alternative treatments for active and chronic liver disease. The formulation of a multifunctional scaffold that has the potential to mimic the complex extracellular matrix (ECM) and their influence on cellular behavior, are essential for culturing cells on a construct. The separate employment of topographic or biological cues on a scaffold has both shown influences on hepatocyte survival and growth. In this study, we investigate both of these synergistic effects and developed a new procedure to directly blend whole-organ vascular perfusion-decellularized rat liver ECM (dECM) into electrospun fibers with tailored surface nanotopography. Water contact angle, tensile test, and degradation studies were conducted to analyze scaffold hydrophilicity, mechanical properties, and stability. The results show that our novel hybrid scaffolds have enhanced hydrophilicity, and the nanotopography retained its original form after hydrolytic degradation for 14 days. Human hepatocytes (HepG2) were seeded to analyze the scaffold biocompatibility. Cell viability and DNA quantification imply steady cell proliferation over the culture period, with the highest albumin secretion observed on the hybrid scaffold. Scanning electron microscopy shows that cell morphology was distinctly different on hybrid scaffolds compared to control groups, where HepG2 began to form a monolayer toward the end of the culture period; meanwhile, typical hepatic markers and ECM genes were also influenced, such as an increasing trend of albumin appearing on the hybrid scaffolds. Taken together, our findings provide a reproducible approach and utilization of animal tissue-derived ECM and emphasize the synergism of topographical stimuli and biochemical cues on electrospun scaffolds in liver tissue engineering.

## 1 Introduction

Liver disease is the leading cause of death among people aged 35–49 in the UK, and its mortality rates are forecasted to exceed that of heart disease in the next few years,^1^ with cases of liver cancer having risen almost two-thirds in the UK in the past decade.^1^ Whole-organ transplantation is the only definitive cure; however, the availability of donor livers is very limited.^2^ There is a shift toward tissue engineering (TE) for the development of novel treatments.^3^ In the last decade, researchers have focused on fabricating multifaceted TE platforms to structurally and physiologically mimic the extracellular microenvironment which supports cells within native tissue.^4−6^ Such platforms include hydrogels, 3D printed scaffolds, electrospun fibers, microfluidic systems (organ-on-a-chip), and decellularized/recellularized tissues.^7−13^ In general, scaffold-based 3D culture systems used for the cultivation of hepatocytes are divided to three groups. One is hydrogel-based scaffolds, using natural-derived biomaterials such as collagen, alginate, gelatin, fibrin, and synthetic polymers such as poly lactic acid (PLA), poly glycolic acid (PGA), polycaprolactone (PCL), polyvinyl alcohol (PVA), and the combination of them.^14^ The second is porous scaffolds such as decellularized matrices and constructs made by lyophilization, 3D printing, and electrospinning.^15,16^ The third is microfluidic devices such as LiverChip and HepaChip, using a continuous medium flow to improve the nutrient and oxygen supply.^17,18^

Within these platforms, cells can be exposed to different topographic structures, which can influence their proliferation, migration, differentiation, and function.^19−21^ In order to mimic part of the complexity in native ECM, scaffolds have been fabricated with various surface features such as pores, wrinkles, and anisotropic structures.^22−27^ However, studies have indicated that cells are influenced not only by microarchitectures but also nanoscale structures.^28−30^ Interestingly, studies on human mesenchymal stem cells (hMSCs) and macrophages revealed that the nanotopography of electrospun fibers influenced cell spreading, morphology, and proliferation.^28,31^ In our previous study, we also demonstrated that electrospun fibers with different sizes of surface nanotopography had an influence on hepatocytes.^32^

Another feature of these platforms is their composition; in healthy tissue, ECM plays a vital role in supporting cell growth and behavior by providing structural, mechanical, and bioactive cues.^33^ In recent years, single and multiple ECM proteins have been incorporated into polymer scaffolds to improve their biocompatibility and function.^34−41^ However, a single protein cannot mimic all functions attributed to whole ECM.^42,43^ Whole-organ decellularized ECM incorporated into artificial scaffolds has shown advantages such as affecting hydrophilicity, cell adhesion, proliferation, and behavior.^33,44−46^ This has also been seen with hepatocytes, with studies showing that rat and human liver dECM can impact cell survival and proliferation.^4,36,47,48^

To date, the focus on scaffold topography and dECM combinations has mainly been investigated on the macroscale.^42,49−52^ However, limited attention has been devoted to the synergistic effects of topographical and biochemical cues on hepatocytes for liver tissue engineering. We have therefore developed a new protocol to incorporate the perfusion of whole-organ decellularized rat liver ECM into electrospun fibers with surface depressions to explore the combined effect on human hepatic carcinoma cells (HepG2). This cell line is a proven tool for analyzing hepatocyte culture platforms due to its availability, phenotypic stability, and high degree of morphological and functional differentiation in vitro.^53−56^

## 2 Methodology

### 2.1 Rat Liver Isolation and Decellularization

The whole liver was harvested from an adult Sprague Dawley male rat (supplied by Charles River) immediately after cervical dislocation, as previously described.^47^ The rat was sterilized with 70% ethanol, and the inferior vena cava was exposed through an abdominal incision. Viscera in the abdominal cavity were then removed. The common bile duct and the distal end of the hepatic portal vein were ligated and transected. A 20-gauge cannula with a 32 mm diameter (Surshield Versatus Winged and Ported IV Cannula) was inserted into the portal vein, and a 50 mL syringe was connected to the cannula to slowly perfuse 100 mL of 0.1% sodium nitroprusside (Sigma) and phosphate-buffered saline (PBS, Gibco) to dilate the vasculature and remove the blood.^57^ Once the liver turned pale in color, the cannula with the portal vein was tightly closed with a thread. The connective tissue around the liver was cut, and the whole liver, including the cannula, was transferred into a homemade decellularization device and perfused with 0.25% sodium dodecyl sulfate (SDS, Sigma) solution through a pump at a flow rate of 5 mL/min. The solution was changed after 4 h and perfused overnight. On the second day, the solution was replaced with ultrapure water, changed after 4 h, and perfused overnight. The decellularized liver was frozen at −80 °C, freeze-dried, and then stored at −80 °C until use.

All animal experiments were approved by the University of Edinburgh Animal Welfare, Ethical Review Body, and the UK Home Office. All experiments with animals were performed at the University of Edinburgh in accordance with the procedural guidelines and severity protocols from the U.K. Home Office Animals Scientific Procedures Act.

### 2.2 ECM Production

The decellularized liver was lyophilized and cut into small pieces, and then 5 mg of dECM was solubilized in 1 mg/mL pepsin (Sigma, 3706 units/mg) in 1 mL of 0.01 M hydrochloric acid (HCL, Sigma). The solubilization was performed for 3 days at room temperature (RT) with constant agitation on a SRT9D roller mixer (Stuart) at 8 rpm. The dECM solution was neutralized by 0.1 M sodium hydroxide (NaOH, Sigma) to a final pH of 7 to stop the digestion. The solution was then lyophilized into a very fine powder, collected, and stored at 4 °C until further use.

### 2.3 Electrospinning Solution Preparation

To make the depression fiber scaffold with dECM (DFECMS), 1 mg (0.07% w/w) and 2 mg (0.14% w/w) of dECM powder were weighed and put into a 3 mL borosilicate glass tissue homogenizer. Two milliliters of chloroform (CFM, Sigma) was added into the homogenizer and manually ground to obtain a suspension solution. The solution was then mixed with 7 mL of CFM, 1 mL of dimethyl sulfoxide (DMSO, Sigma), and 1.4 g of PCL pellets (*M*_n_ = 80,000 Da, Sigma). The total final solution was 10 mL. The polymer was dissolved overnight at RT with agitation on a roller mixer at 8 rpm. The depression fiber scaffold (DFS) was made using the previous described polymer solution without dECM. The smooth fiber scaffold (FS) was produced by dissolving 1.6 g of PCL into CFM/methanol (Sigma) (5:1). The parameter properties of the four types of fibers are shown in Table 1.

**Table 1:** Electrospinning Solution Parameters: Fiber Scaffold (FS), Depression Fiber Scaffold (DFS), Depression Fiber Scaffold with 0.07% ECM (0.07% DFECMS), and Depression Fiber Scaffold with 0.14% ECM (0.14% DFECMS)

	FS	DFS	0.07% DFECMS	0.14% DFECMS
ECM content			1 mg	2 mg
PCL content	1.6 g	1.6 g	1.4 g	1.4 g
DMSO		1 mL	1 mL	1 mL
CFM	8.4 mL	9 mL	9 mL	9 mL
methanol	1.6 mL			

### 2.4 Electrospinning

Electrospinning was performed using a syringe pump EP-H11 (Harvard Apparatus), an EC-DIG electrospinning system (IME technologies) at RT, and a metal mandrel covered by a layer of aluminum foil to collect the fibers. The electrospinning parameters are shown in Table 2. The collected fiber sheets were dried for 2 days in a fume hood and stored at 4 °C until use.

**Table 2:** Electrospinning Parameters

volume per hour	total volume	positive voltage	negative voltage	Mandrel needle distance	Mandrel rotation speed	needle diameter
5 mL	10 mL	14 kV	–4 kV	23 cm	250 rpm	0.8 mm

### 2.5 Contact Angle Measurements

The contact angle was measured on dry scaffolds. A 5 μL water drop was pipetted onto the scaffold surface; the images were captured using a DMK 41 AU02 monochrome 1280 pixel × 960 pixel camera at 5 Hz every 0.2 s. The measurements were conducted with the software ImageJ and the plugin Contact Angle (as shown in Figure 2E), *N* = 5.

### 2.6 Mechanical Testing

The sample was prepared by cutting the scaffolds into rectangular strips with a scalpel (gauge length: 20 mm, width: 5 mm). An Instron 3367 tensile testing machine (Instron, UK) with a 50 N load cell was used to test the tensile properties as previously described.^32^ Briefly, all samples were subjected to monotonic tensile loading at a strain rate of 50% ε min^–1^ until failure (*N* = 5). The ultimate tensile strength and Young’s modulus were calculated. The incremental Young’s modulus was taken at various strain bands: 0–5%, 5–15%, 15–25%, and 25–35%.

### 2.7 Scanning Electron Microscopy

Scaffolds were sputter-coated with gold–palladium using an Emscope SC500A sputter coater before SEM. Scaffolds were visualized using a Hitachi TM4000 SEM (Hitachi) with a 15 kV accelerating voltage and a mixed-sensor mode combining backscattered and secondary electron detectors at different magnifications. The fiber diameter and depression size were determined from the images using ImageJ software.

### 2.8 In Vitro Degradation Evaluation

The samples were cut into rectangular strips with a scalpel (gauge length: 20 mm, width: 5 mm) and weighed for the initial dry weight *W*_o_. Then, these scaffold strips were sterilized with 70% ethanol for 15 min and a further 30 min in a fresh ethanol solution in a bio-hood. They were then washed with sterilized PBS three times for 10 min each. The scaffolds were then incubated in 5 mL of serum-free media containing 1% Anti-Anti at 37 °C. After 14 days, the samples were removed from the media and washed in deionized water for three times for 10 minutes each and dried in a fume hood for 24 h. The samples were weighed again as the dry weight after degradation *W*_d._ The weight loss as a percentage of the initial weight was calculated according to eq 1. The physical appearance of the samples after degradation was examined by JEOL JSM-IT100 SEM (JEOL Ltd., Japan) with a 15 kV accelerating voltage at different magnifications. Further analysis was done with ImageJ software. The mechanical property of the samples after degradation was quantified according to Section 2.6.
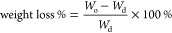
1

### 2.9 Scaffold Preparation

Ten millimeter scaffold discs were punched out via a biopsy punch, soaked in 70% ethanol until the aluminum foil could be removed, and soaked again for a further 30 min in a fresh ethanol solution for sterilization. They were then washed in sterilized PBS three times for 10 min each in the bio-hood. The scaffolds were then incubated (5% CO2 and 37 °C) overnight in serum-free media containing 1% antibiotic/antimycotic (Anti–Anti) and then separated into 24-well plates for cell seeding.

### 2.10 Seeding Cells and Culture

HepG2 cells (Sigma) at passage 8 were cultured for a week until 70% confluency. Cells were then trypsinized from the tissue culture flasks by a 5 min incubation in 3 mL of trypsin-ethylenediaminetetraacetic acid (EDTA) (Sigma) and counted using the Trypan Blue method.^58^ Cells were suspended in 1.5 mL of complete Eagle’s minimum essential media (MEM, Gibco) supplemented with 1% l-glutamine (Gibco), 1% non-essential amino acids (NEAA, Sigma), and 10% fetal bovine serum (FBS, GE Healthcare), and 20 μL of cell solution (1× 10^4^ cells) was seeded directly onto each scaffold. The cells were allowed to adhere for 2 h in a 5% CO_2_ incubator set to 37 °C. Then, an additional 1.5 mL of complete media was added to each well. The media was changed every 48 h, and samples were subsequently collected at 24 h, 7 days, and 14 days.

### 2.11 Cell Viability Assay

A CellTiter-Blue assay (Promega) was performed at each time point as per the manufacturer’s instruction. Briefly, the scaffolds were transferred to new well plates, and 500 μL of the mixed solution of CellTiter-Blue reagent:fresh media (1:4) was added to each well and covered with aluminum foil to protect samples from light. One hundred microliters of incubated solution from each well was placed into a black well plate; the measurements were taken from a CLARIO-star Plus microplate reader (BMG LABTECH) after 3.5 h of incubation at an excitation wavelength (ex) of 525 nm and an emission wavelength (em) of 580–640 nm. A negative control, without cells, was used to determine the background fluorescence.

### 2.12 DNA Quantification

This assay was performed using a Quant-IT Picogreen dsDNA assay (Promega) as per the manufacturer’s protocol. Samples were lyophilized and digested in a papain solution containing 2.5 units of papain, 5 mM cysteine HCL, and 5 mM EDTA in DNA free water (all Sigma). Scaffold digestion lasted for 24 h at 65 °C, and tissue (ECM) digestion lasted for 48 h with periodic mixing using a vortexer. The digested solution was added with an aqueous working solution of the Quant-iT into a black 96-well microplate. The measurements were taken after 5 min of incubation at RT, and the results were read at ex 480 nm/m 510–570 nm in the CLARIO-star Plus microplate reader (*N* = 5).

### 2.13 Albumin Quantification

A bromocresol green albumin assay (BCG, Sigma) was performed to quantify the production of albumin by HepG2 cells over 72 h according to the manufacturer’s protocol. Briefly, fresh cell culture media was added at the day 7 timepoint and exposed to the scaffolds for 3 days (72 h) and collected at day 10. The samples were added with BCG solution and incubated in a clear-bottom 96-well microplate at RT for 5 min. Results were read at an absorbance of 620 nm in the CLARIO-star Plus microplate reader (*N* = 5).

### 2.14 Fluorescent Staining

The cell-seeded scaffolds were immersed in 10% formalin (Sigma) on each timepoint for 30 min at RT and then washed with PBS three times and stored in PBS at 4 °C before staining. The scaffolds were then permeablized with 0.2% Triton X-100 (Sigma) in PBS for 10 min before three rounds of PBS washing. Then, each sample was stained with 300 nM 40,6-diamidino-2-phenylindole (DAPI, Thermo-Fischer) in PBS for 15 min and washed thrice in PBS for 5 min each at RT. Samples were then stained with 1 μL/mL fluorescent-conjugated Phalloidin 514 (Sigma) in 1% bovine serum albumin (BSA, Sigma) in PBS for 30 min and then followed by three rounds of washing at RT. Stained scaffolds were kept in PBS, wrapped in foil, and stored at 4 °C until imaged. A Zeiss Axio Imager fluorescence microscope was used to take the images. The images were processed using Icy software.

### 2.15 Immunohistochemical Staining

To determine the presence of ECM proteins, the scaffolds were stained with 1 μL/mL primary antibodies from rabbits for collagen I (Stratech) and fibronectin (Sigma) in 1% BSA in PBS overnight at 4 °C. On the next day, those samples were stained with 1 μL/mL fluorescently conjugated rabbit IgG secondary antibodies (Abcam) in 1% BSA in PBS at RT for 1 h according to the manufacturer’s protocol (Abcam).

To evaluate protein production by HepG2, the formalin-fixed cell-seeded scaffolds (as described in Section 2.13) were stained with E-cadherin (Stratech) primary antibody overnight at 4 °C and secondary antibody to complete the staining as the manufacturer’s protocol (Abcam). All immunostaining images were obtained using the Zeiss Axio Imager fluorescence microscope and processed using Icy software.

### 2.16 Osmium-Staining SEM

The osmium-staining procedure was performed as previously described.^32^ Briefly, the cell-seeded scaffolds were immersed in 4% glutaraldehyde (Sigma) for 30 min at RT on each timepoint and then washed with PBS three times and stored in PBS at 4 °C until use. Glutaraldehyde-fixed cell-seeded scaffolds were incubated in 0.1% osmium tetroxide (TAAB) in deionized water for 30 min before dehydrating in ethanol (Sigma) and hexamethyldisilazane (HDMS, Sigma). Samples were dried overnight in the fume cupboard and visualized using a Hitachi TM4000 SEM (Hitachi) at different magnifications.

### 2.17 Reverse Transcription Polymerase Chain Reaction

The cell-seeded samples (*N* = 5) were placed in 500 μL of Tri-Reagent (Sigma) and stored at −80 °C. RNA was extracted from the thawed samples using CFM and purified using Qiagen’s RNeasy spin column system as per a previously described method and the manufacturer’s instructions.^47^ Nanodrop (Thermo Scientific NanoDrop 2000c spectrophotometer) was used to measure the nucleic acid concentration of each sample, and then RNA was reversed-transcribed to complementary DNA (cDNA) using reverse transcriptase (Promega) according to the manufacturer’s instructions. Quantitative real-time polymerase chain reaction (qRT-PCR) was performed using a LightCycler1 480 Instrument II (Roche Life Science). The relative amount of target RNA expression in the scaffold was quantified by the 2^–ΔΔCt^ method. Gene expression results were measured relative to glyceraldehyde 3-phosphate dehydrogenase (GAPDH) (housekeeping gene) and normalized to the smooth PCL scaffold (FS) at 24 h.

### 2.18 Statistical Analysis

Statistical analysis was performed by Minitab software. The mean and standard deviation were calculated. The minimum biological replication is *N* = 3 (RT-PCR), and the test used in this study was one-way ANOVA with Turkey’s post hoc test. Statistical significance was displayed as *p < 0.05, **p < 0.01, and ***p < 0.001 unless labeled with other symbols.

## 3 Results

### 3.1 Scaffold Properties

**Figure 1:**
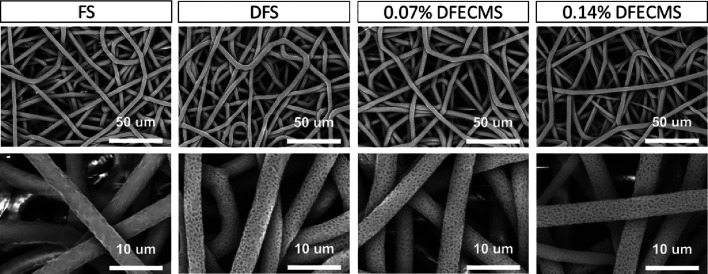
SEM images of all types of scaffolds (small magnification: ×2000, scale bar = 40 μm; large magnification: ×10,000, scale bar = 10 μm).

All scaffolds showed similar fiber diameters (roughly 4 μm), and the depression diameters ranged from 133 to 163 nm (Figure 1 and Table 3). The fibers all showed a random electrospun morphology with no alignment visible. The maximum and minimum fiber sizes were seen in 0.14% DFECMS at 2.3 to 5.8 μm. The smallest size variation was seen in the FS going from 3.5 to 4.74 μm. In the depression size, the largest variation was seen in the 0.07% DFECMS with the smallest seen in the 0.14% DFECMS with values of 15.47 and 7.7 nm, respectively.

**Table 3:** Fiber and Depression Diameters

	FS	DFS	0.07% DFECMS	0.14% DFECMS
fiber diameter (μm)	4.061 ± 0.28	3.9 ± 0.36	3.88 ± 0.48	4.2 ± 0.7
min	3.5	3.22	2.98	2.3
max	4.74	4.52	4.67	5.8
depression diameter (nm)		149.06 ± 14.59	146.72 ± 15.47	140.94 ± 7.7
min		134.8	135.13	130.8
max		167.68	162.61	154.51

**Figure 2:**
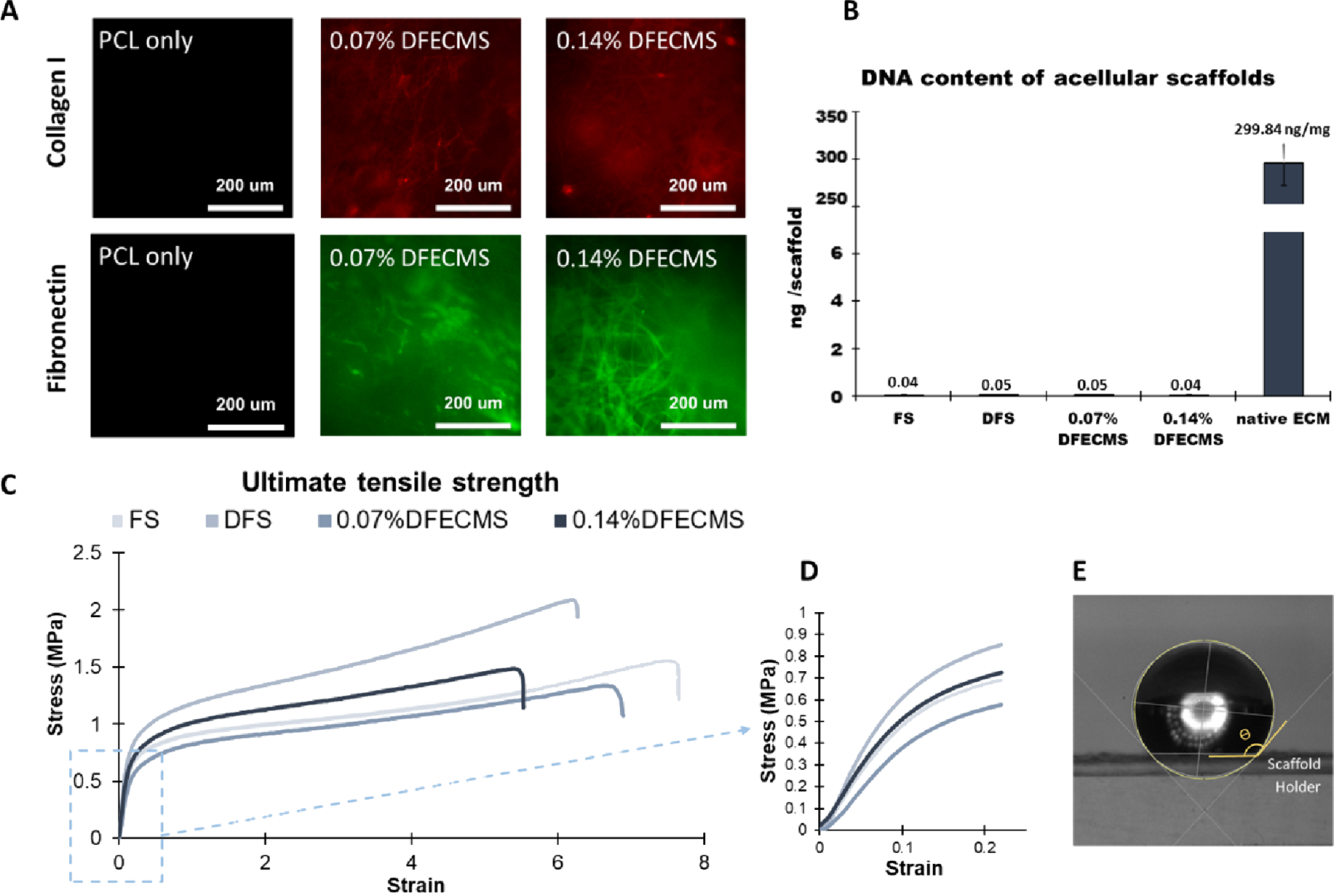
(A) Collagen I and fibronectin staining confirmed the presence of dECM on the fiber at 25× magnification; (B) DNA quantification of the acellular scaffolds and native tissue; (C) ultimate tensile strength of each type of scaffold; (D) graph of stress versus strain of the scaffolds; (E) illustration of the measurement of contact angles.

Collagen I and fibronectin staining of the scaffolds confirmed the presence of dECM in the fibers, as shown in Figure 2A. This indicates the retention of collagen and fibronectin proteins through decellularization and electrospinning processes. Figure 2B shows that no DNA was detected from all scaffolds (before seeding cells) compared to the native liver ECM, which has a DNA content of 299.84 ± 29 ng/mg.

Tensile testing (Figure 2C,D and Table 4) showed that DFS had a significantly higher ultimate tensile strength (UTS) than FS, 0.07% DFECMS, and 0.14% DFECMS (*P* < 0.001). Also, the Young’s modulus of DFS was significantly higher than that of the other scaffolds at 0–5% strain band and significantly higher than 0.07% DFECMS at the 5–15% strain band. There was also a significant difference between 0.07% DFECMS and 0.14% DFECMS. The elongation at break showed that DFS had a 14% (*P* < 0.05) reduction compared to FS, and 0.14% DFECMS had a 24% (*P* < 0.001) reduction compared to that of FS. Contact angle measurements showed how incorporating depression and dECM significantly increased the hydrophilicity of the scaffolds. The hybrid scaffolds have about 15 and 8% lower contact angles than that of FS and DFS, respectively (*P* < 0.05) (Table 4).

**Table 4:** Mechanical Properties of Each Scaffold^a^

	FS	DFS	0.07% DFECMS	0.14% DFECMS
contact angle (1s)	121.13 ± 0.98^γ^	112.87 ± 0.73^γ^	102.97 ± 1.82	104.9 ± 1.12
ultimate tensile strength (MPa)	1.56 ± 0.04	2.09 ± 0.36	1.34 ± 0.17	1.49 ± 0.07
elongation at break %	768 ± 17.11	658.7 ± 48.43	690.67 ± 30.28	582.16 ± 36.3
Young’s modulus at strain 0–5% (MPa)	5.28 ± 0.41^α^	7.07 ± 1.46^αβ^	3.69 ± 1.24^β^	5.88 ± 0.63^β^
Young’s modulus at strain 5–15% (MPa)	4 ± 0.08	4.98 ± 0.91^α^	3.27 ± 0.45^α^	4.22 ± 0.24
Young’s modulus at strain 15–25% (MPa)	2.85 ± 0.04	3.53 ± 0.64	2.4 ± 0.3	3.01 ± 0.18
Young’s modulus at strain 25–35% (MPa)	2.18 ± 0.02	2.74 ± 0.48	1.88 ± 0.24	2.34 ± 0.13

a Contact angle results with the symbol γ indicate a *P* < 0.001 significant difference from all other groups; Young’s modulus results share the same symbol α, β indicate a *P* < 0.05 significant difference from each other at the same strain band.

### 3.2 Scaffold Degradation Study

**Figure 3:**
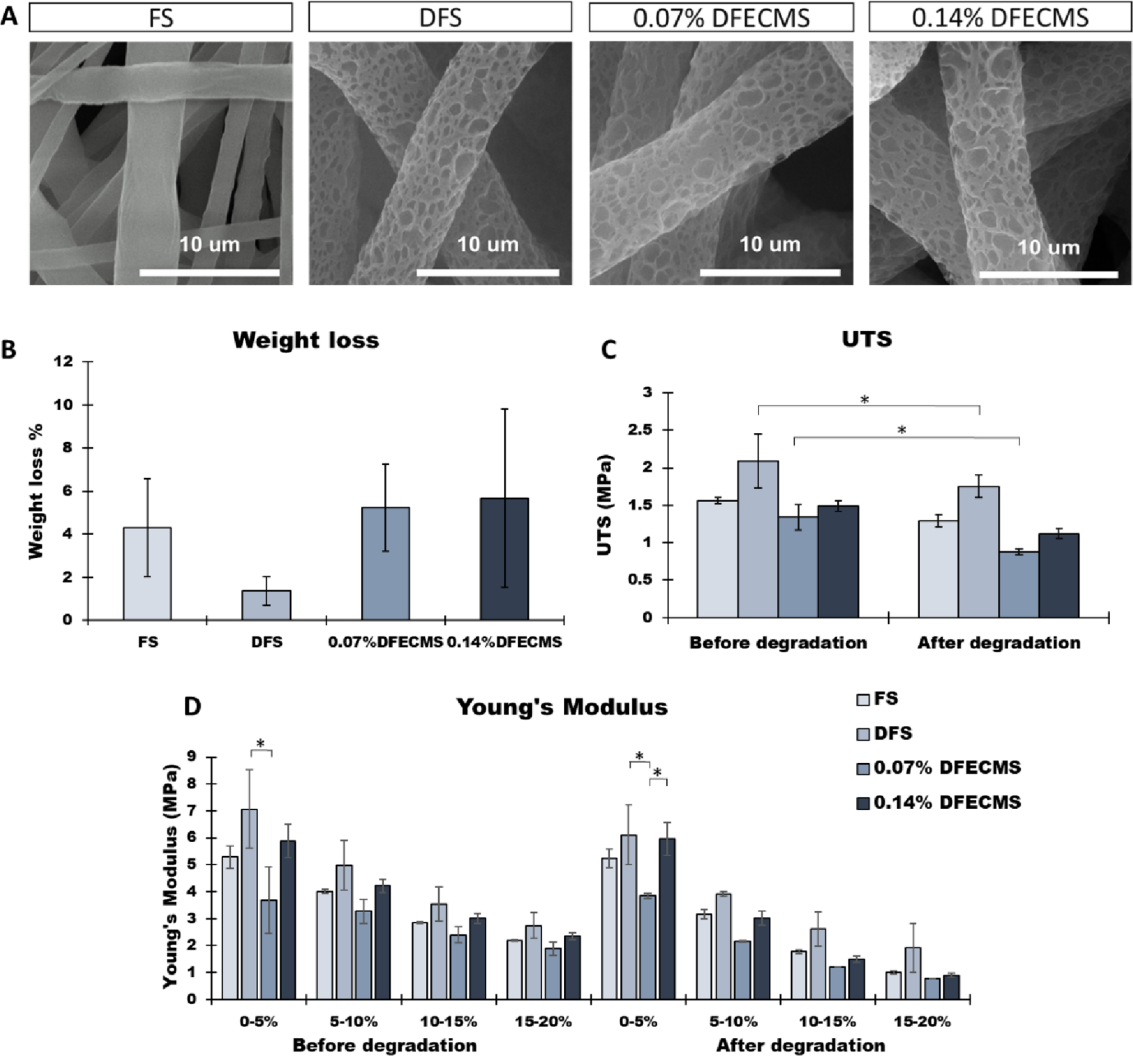
(A) SEM images of each type of fiber scaffold after degradation, ×10,000. (B) Weight loss % after degradation. (C) Ultimate tensile strength (UTS) of each scaffold before and after degradation. (D) Incremental Young’s modulus of each scaffold before and after degradation at various strain bands. *P < 0.05.

A scaffold degradation study was conducted to analyze the stability of the scaffolds’ fiber topography, weight loss, and mechanical properties over time in culture media. As shown in Figure 3A, the fiber topography did not change after 14 days of incubation at 37 °C. The weight loss percentage (%) results (Figure 3B) showed no significant difference between each scaffold; however, the 0.07 and 0.14% DFECMS showed higher average weight loss % compared to FS and DFS, reaching 5.24 and 5.66%, respectively. Mechanical testing showed that the UTS of all scaffolds reduced after degradation, the significant difference being observed on both DFS and 0.07% DFECMS (Figure 3C). The Young’s modulus of all the scaffolds slightly decreased after degradation (Figure 3D). The consistency can be observed such that the Young’s modulus of DFS was higher than 0.07% DFECMS both before and after degradation.

### 3.3 Cell Analysis

**Figure 4:**
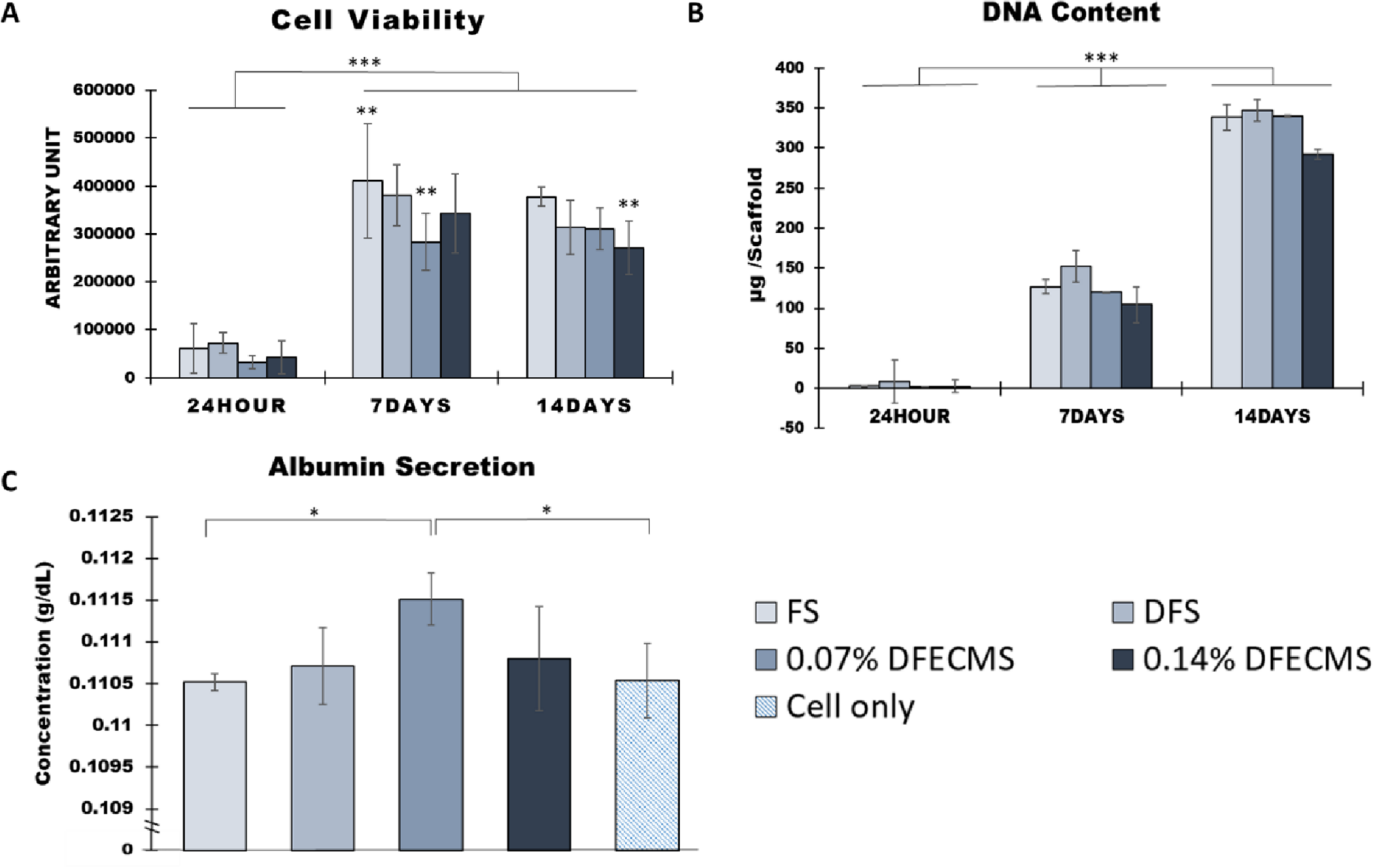
Cell analysis of different assays: (A) cell viability; (B) DNA content; (C) albumin secretion for 72 h between day 7 and day 10. *P < 0.05, **P < 0.01, ***P < 0.001.

CellTiter-blue results (Figure 4A) show that cell viability at 7 and 14 days was significantly higher than 24 h for all groups (*P* < 0.001). FS on day 7 has the highest average cell viability. The average cell viability of FS, DFS, 0.07% ECMDF, and 0.14% ECMDF significantly increased by 84, 80, 88, and 88% respectively from 24 h to day 7 (*P* < 0.001). There was also a significant difference on day 7 between FS and 0.07% ECMDF (*P* < 0.01). Also, there was a significant difference between day 7 FS and day 14 0.14% DFECMS (*P* < 0.01).

DNA quantification results (Figure 4B) show that the DNA content of all the four scaffolds steadily increased during the culture period, and significant differences were observed between timepoints. All groups show significantly increased levels of 97% on average between 24 h and day 7. There was a further increase by 62, 56, 64, and 65% for FS, DFS, 0.07% ECMDF, and 0.14% ECMDF, respectively, between day 7 and day 14. There were no significant differences observed between the scaffold groups at single timepoints.

Hepatic function was accessed using albumin secretion between day 7 and day 10 (72 h) (Figure 4C). The 0.07% DFECMS scaffold showed a significantly higher albumin concentration (0.1115 ± 0.0003 g/dL) than FS and cells only (cultured on the tissue culture plastic) (*P* < 0.05), with an average difference of 0.001 g/dL.

### 3.4 Osmium Staining

**Figure 5:**
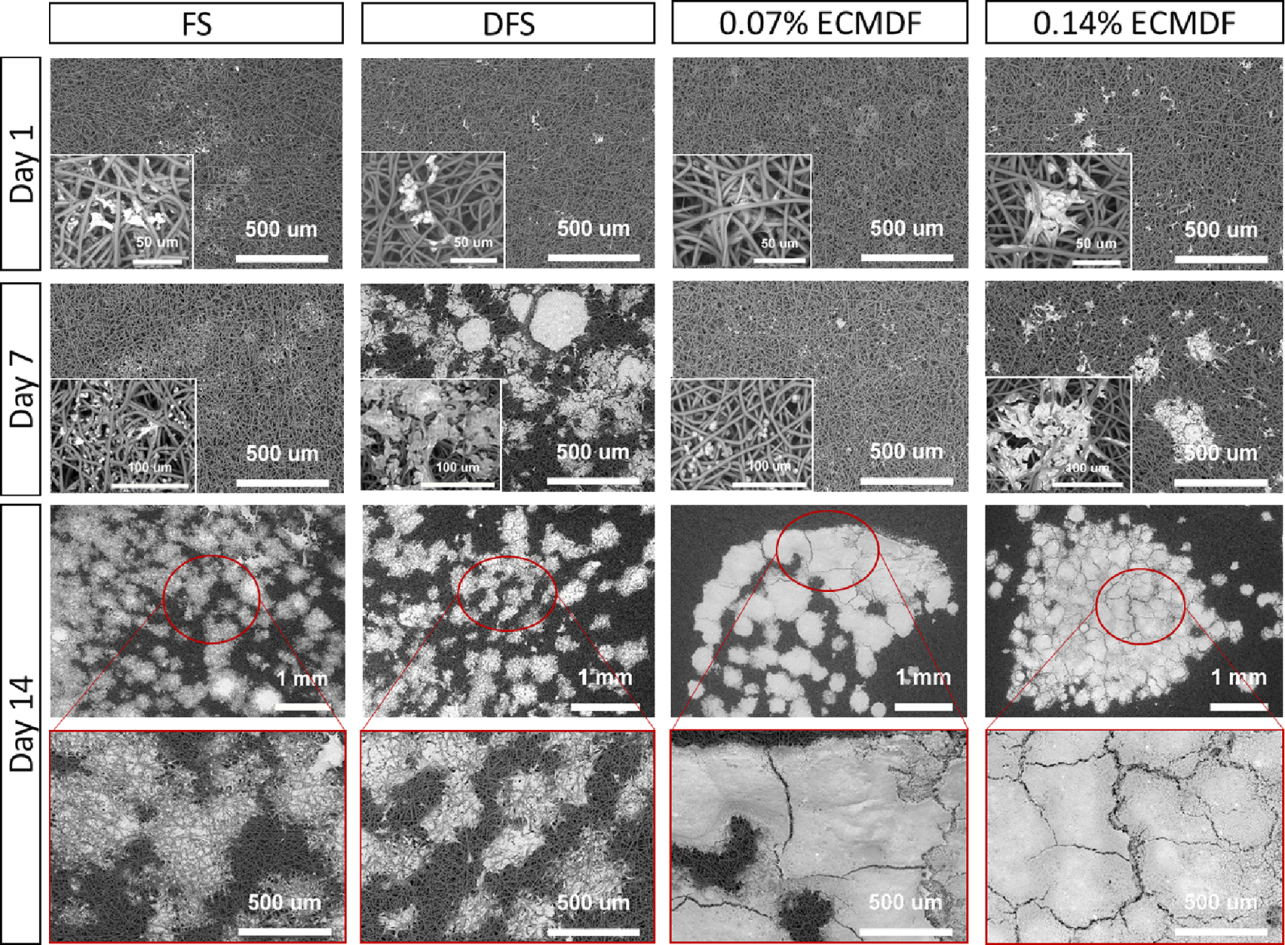
SEM images of osmium staining of the cell-seeded scaffolds for all timepoints.

Representative images (Figure 5) of the osmium-stained scaffolds showed cell attachment and morphology. It was noted that cells were attached to the scaffolds at 24 h and grew steadily to day 14. Interestingly, on day 14, the cellular aggregates tended to be more widely distributed throughout the scaffolds without dECM. In contrast, the dECM scaffolds show more densely packed aggregates, which form a monolayer and have more cell–cell interactions on the hybrid scaffolds.

### 3.5 Fluorescence Staining and Gene Expression

**Figure 6:**
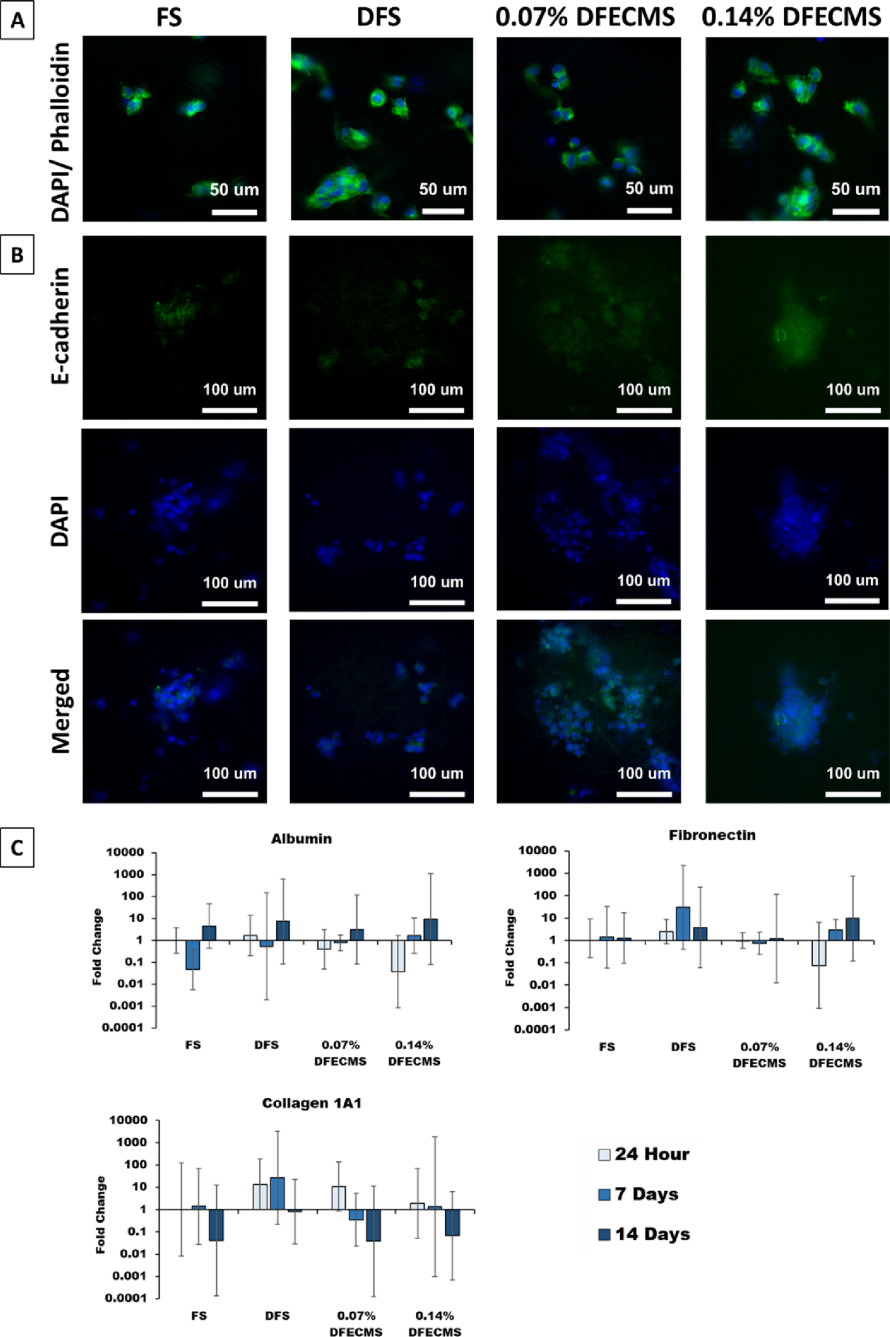
(A) DAPI /phalloidin (blue = nucleus, green = actin filament) staining of cells on 24 h culture; the cells show a round appearance at all scaffolds. A 40× magnifying objective was used to take pictures, and the white scale bar = 50 μm. (B) Immunostaining of cells on all scaffolds at 24 h (green = E-cadherin, blue = nucleus); a 40× magnification objective was used to take pictures, and the white scale bar = 100 μm. (C) RT-qPCR gene expression of albumin, fibronectin, and collagen 1A1 over 14 days; all results relative to the housekeeping gene GAPDH and normalized to FS at 24 h.

At 24 h, DAPI and phalloidin staining showed that all scaffold types produce a similar cell morphology (Figure 6A). Immunostaining of HepG2 on the different scaffold types indicated that they all promote E-cadherin protein expression (Figure 6B). DAPI stains highlighted the cell nuclei on the scaffolds (Figure 6B).

Gene expression analysis (Figure 6C) shows the fold change of albumin, fibronectin, and collagen 1A1 relative to FS at 24 h. Hybrid scaffolds showed upward trends for albumin and fibronectin over 14 days, and all groups are showing upregulation of albumin and fibronectin on day 14, though large variations were observed. In particular, within 24 h, the main albumin expression of 0.07% DFECM and 0.14% DFECM was 2.5-fold and 26-fold lower than that of FS, respectively; on day 14, it was 3.2-fold and 9.3-fold higher than the control, respectively. In terms of fibronectin expression, 0.14% DFECM decreased 13-fold at 24 h and increased 9.6-fold on day 14. Whereas small fold changes were observed in both FS and 0.07% DFECM groups, only 1.3-fold and 1.2-fold upregulation were observed on day 14, respectively. Interestingly, the highest level change observed in DFS was within a 30.4-fold increase on day 7, while it reduced to 3.8-fold on day 14. Conversely, a downward trend was noted on collagen 1A1 over the culture period, and all groups showed downregulation on day 14, though there was no significant difference found between groups. Notably, DFS showed the highest upregulation with a 26.6-fold increase on day 7, and 0.07% DFECM showed the highest downregulation with a 26.3-fold decrease on day 14.

## 4 Discussion

The presented research work studies the formulation of surface depressions on electrospun PCL fiber scaffolds that contain rat dECM. For this purpose, three types of scaffolds with similar surface nanodepressions (145 nm) were presented with all groups having similar fiber diameters (4 μm) and morphologies. The approach used to create these fibers with surface depressions is a solvent CFM/non-solvent DMSO phase-separation system. PCL, a commonly used biomaterial in electrospinning, is soluble in CFM but insoluble in DMSO.^59^ Due to the high boiling point of DMSO (189 °C), which is three times higher than CFM (61 °C), it evaporates first during the spinning process. This forces the ratio of solvent and non-solvent to change quickly and induces phase separation, resulting in surface depressions on the scaffolds.^60^

The addition of rat liver dECM made the PCL fiber morphology unstable in this solvent/non-solvent system. The concentrations (0.07% w/w dECM, 0.14% w/w dECM) selected in this study as concentrations greater than 0.2% w/w were found to induce inconsistent morphology and topography during the method development (results not shown). We maintain the nanodepressions and consistent morphology by digesting and varying the amounts of dECM, solvent mixing processes, polymer concentration. and spinning parameters. dECM is water-insoluble, and only a few solvents can be used to dissolve it for electrospinning purposes.^61^ Hexafluoroisopropanol (HFIP) and 1,1,1,3,3,3-hexafluoro-2-propanol (HFP) is one of the mainly used solvents for ECM dissolution in electrospun scaffolds.^4,47,62−65^ However, in our study, in order to create the surface depression structure, different solvent systems (CFM/DMSO) needed to be used. dECM is poorly soluble in CFM because components like collagen and elastin are hard to break down due to their cross-linking of amino acid chains.^66,67^ Our method of solubilizing dECM enzymatically in pepsin/HCL provides a favorable way to break down the insoluble components into smaller peptides.^68,69^ Enzymatic pepsin digestion in an acidic buffer is the gold standard in solubilizing dECM, though the process can cause the loss of integrity to some ECM components.^70,71^ Furthermore, the homogenization provided a good mechanical force to break down the big junctions left in the digested dECM, which was blended with PCL in our solvent system. Immunostaining for collagen I and fibronectin in the scaffolds confirmed that dECM has been incorporated (Figure 2A).

Tensile testing showed the mechanical changes of using non-solvent (DMSO) and adding dECM in the manufacture of electrospun fibers. The Young’s modulus of DFS was 34% higher than FS and previous studies, including results from our lab, showing that adding DMSO can increase the mechanical stiffness of the electrospun fibers.^32,72^ Some of these differences may be attributed to DMSO changing the crystalline region and molecular orientation of PCL, which could partly contribute to the change in tensile properties.^73^ The inclusion of dECM decreased key aspects of the material mechanical properties. In particular, the DFS group UTS in the 0.07% DFECMS and 0.14% DFECMS scaffolds decreased by 36 and 29%, respectively, and the Young’s modulus also respectively decreased by 48 and 17% in the 0–5% strain band. This reduction of tensile strength due to the addition of dECM has been shown in the literature.^62,74^ Conversely, studies have also shown that dECM can increase the ductility of polymer scaffolds due to it having a higher elongation at break.^62,75^ Our results show that including DMSO in the electrospinning polymer solution leads to higher stiffness, while adding dECM leads to a reduction of stiffness. In vivo, the metabolic state of hepatocytes is influenced by tissue stiffness, as is observed during fibrosis-induced mechanical changes within the liver microenvironment.^76^ Studies have shown that key hepatocyte functions such as cytochrome P450 activity and albumin production are significantly reduced with increasing substrate stiffness.^77−80^ Xia et al. demonstrated that increasing the hydrogel substrate stiffness upregulated membrane integrin expression and caused nuclear relocation of membrane bound β-catenin in L-02 cells, indicating a shift in cell–cell and cell–matrix adhesion patterns that may be related to the observed changes in hepatocyte function.^81^ The stiffness of the scaffolds described in the present study fall in the range of MPa, which is considerably higher than that of liver tissue (healthy liver to fibrotic liver: 4.5–37 KPa) but is lower than that of collagen fibrils.^81,82^ Future efforts should confirm how electrospun fiber stiffness measurements, at different scales, relate to molecular mechanotransduction pathways in hepatocytes and in which conditions this can significantly influence hepatocyte metabolic function.

Water contact angle measurements showed that depression and hybrid scaffolds both have improved hydrophilicity. In our results, after the incorporation of surface depressions, the angle was significantly reduced by 7.4% compared to FS. Further reduction was found when dECM was incorporated, where the angle was reduced by 18%. Thus, surface depressions and dECM synergistically improved the scaffold’s hydrophilicity. This phenomenon of increased hydrophilicity by adding dECM has previously been shown.^62−64^

PCL is a hydrophobic semi-crystalline polymer with a slow degradation rate (2–4 years) and thus provides good stability.^83,84^ A recent hydrolytic degradation study confirmed there was no significant effect on the morphology of PCL electrospun fibers after 90 days of degradation.^84^ However, studies have shown PCL fibers degrade faster in enzymatic media and in vivo conditions as well as blending with other biomaterials such as PLA, PLLA, chitosan, gelatin, and cellulose, and faster degradation can also be achieved by surface modification.^84−90^ Our results show that fiber topography was maintained after a 14 day incubation in culture media with slight reductions in weight, mechanical stiffness, and UTS. This demonstrates the stability of our system over short-term culture periods in hydrolytic conditions. The long-term degradation effects on these platforms should be obtained to provide a more comprehensive understanding of the changes in scaffold morphology and topography over time. This is important when considering models of tissue injury and disease as well as for regenerative applications in vivo.

Increases in cell viability and DNA content over 7 days of culture indicate that HepG2 hepatocytes were highly proliferative on all scaffolds. Significant increases of DNA were observed on all scaffolds across all timepoints, while cell viability remained consistent between day 7 and day 14. This demonstrated that our scaffolds have the capability to maintain cell attachment and growth. Notably, the CellTiter-blue assay result is not congruent with Picogreen DNA quantification. This could indicate a reduction in metabolic activity or increased cell death between 7 and 14 days of culture. However, differences in the methods of data extraction and validation and possible assay saturation could be responsible for the discrepancy. While we did see increases within the culture periods, we did not see differences between groups. A contradictory finding has been shown previously, where we found a statistical increase in viability in the dECM scaffolds compared to the control group.^47,63,91^ This in part may be explained by the comparatively decreased protein addition in this current study, incorporating an amount 10 times lower.

qRT-PCR results showed that the HepG2 seeded scaffolds expressed albumin, fibronectin, and collagen 1A1, which are key markers in hepatocyte function. In particular, albumin, which is involved in the transport of various hormones, enzymes, and vitamins and maintains an appropriate osmotic pressure of blood,^92^ had an increasing trend on both 0.07% DFECMS and 0.14% DFECMS. Our findings agree with previous research where the addition of dECM into the PCL electrospun fiber scaffold improved the production of albumin over time.^4,47,93^ The increasing trend might also occur due to the increased cell–cell interaction on both dECM scaffolds (Figure 4), as suggested previously.^94^ In addition, apart from albumin gene expression, we also looked at protein production. The results showed significantly higher albumin production in the hybrid scaffold (0.07% DFECMS) compared to the FS and DFS. We also looked at fibronectin gene expression, which showed that the 0.14% DFECMS showed a similar upward trend over 14 days culture, although no significant differences were present. This is a really important gene as it guides cell-adhesive interactions and plays a major role in cell adhesion, growth, migration, and differentiation.^95,96^ In our findings, we see that the 0.14% DFECMS and DFS appear higher than the other groups. These results are similar to previous studies incorporating dECM, which also showed an altered fibronectin expression.^47,97^ Examining collagen 1A1 expression showed a decreasing trend on both hybrid scaffolds as well as a decrease in all groups on day 14. Similarly, this is a critical factor in the liver; however, the overproduction of collagen I is associated with the development of fibrosis.^98−100^ Though statistically significant differences were not found, similar results were observed in previous studies,^4,47^ suggesting that the suppression of collagen 1A1 may be associated with cell metabolic stability after 7 days in culture.^47^

SEM of cell seed scaffolds using osmium staining showed a high amount of cell–cell interaction on both of the DFECMS with the formation of a partial monolayer, while greater spreading was observed for the FS and DFS scaffolds. This increase in cell–cell interaction with the inclusion of dECM has been reported previously.^4,94^ Incorporation of dECM within the scaffold materials can provide ECM adhesion motifs, such as Arg–Gly–Asp (RGD) (collagen, fibronectin) and Tyr–Ile–Gly–Ser–Arg (YIGSR) (laminin).^101,102^ They participate in the regulation of cell adhesion, proliferation, and migration by direct interaction with cell receptors, such as integrins and cadherins.^101−103^ Previous studies have indicated hepatocytes can be organized as a monolayer and anchored tightly to exhibit tight cell–cell interactions on a substrate coated with ECM proteins.^63,102,104^ A study into the specific molecular interactions between HepG2s and the scaffold is necessary to determine how these differences observed in cell spreading kinetics could be driven by factors within the dECM. In relation to the cell-free portion, this is largely due to the seeding approach, where a large droplet is added to the surface of the scaffold for a 2 h period before being flooded with media. This phenome may be removed by altering the seeding methods, but it has no major influence on the current findings.

While this study sheds light on the potential of combining topographical features and dECM, it is not without some limitations. In particular, the cell type chosen in this study was an immortalized cancer cell line. While HepG2s have been shown to be a reliable and relatively stable system to show specific liver functions, they do not fully reflect the primary cell’s behavior. Primary cells have been shown to quickly lose viability and function in vitro, making them an unreliable source to test new biomaterial platforms.^105^ Future studies are needed to evaluate primary hepatocytes to verify the true reflections of primary culture and gain more insight into the influence of the developed scaffolds. Although, so far, the evidence indicates nanopores and roughness on fiber surface can improve cell adhesion and proliferation, the quality of evidence varies across studies.^14^ More detailed studies focusing on specific cell types, polymers, and cells could provide further insights into the mechanisms of topography on a scaffold. Another limiting factor was only investigating blending for the addition of the dECM while there are numerous approaches such as emulsion and coaxial spinning, which can alter the protein location and influence the materials’ characteristics.^34,106^ Additionally, the base fiber size was constant at 4 μm as we needed a stable platform to showcase our additions; however, it is worthy to acknowledge that previous work has shown that hepatocytes react to morphological changes in electrospun scaffolds.^5^ Furthermore, while we used rat liver as the source of dECM, there is potential for using other sources such as human liver and recombinant proteins. While these shortcomings are important considerations, they also highlight the potential of this approach in biomaterial design by the combination of two critical design factors.

## 5 Conclusions

In this study, electrospun PCL fiber scaffolds incorporated with nanoscale surface topography and rat dECM in different concentrations were successfully fabricated for liver tissue engineering for the first time. The synergistic effects of topographical and biochemical cues were further investigated by culturing HepG2 cells. The results indicate that this hybrid scaffold may facilitate cell–cell and cell–material interactions, demonstrating their feasibility for tissue engineering and regenerative medicine. Finally, our study showcases yet another way of developing a controllable and replicable electrospun system with the potential to advance platforms for liver tissue engineering.

## Notes

The morphology and composition of electrospun fiber scaffolds have effects on cell behavior. Investigations into topographical cues, biochemical cues, and their synergism in scaffolds have shown benefits for culturing cells in vitro. Herein, our findings provide a new approach that enables the inclusion of dECM into electrospun fibers with tailored surface depressions while maintaining a consistent morphology. Our results suggest that the combination of topography and dECM may facilitate cell–cell and cell–material interactions, thus promoting the feasibility of fiber scaffolds in liver tissue engineering.
